# Evaluation of Xpert MTB/RIF for Detection of Tuberculosis from Blood Samples of HIV-Infected Adults Confirms Mycobacterium tuberculosis Bacteremia as an Indicator of Poor Prognosis

**DOI:** 10.1128/JCM.00330-13

**Published:** 2013-07

**Authors:** Nicholas A. Feasey, Padmapriya P. Banada, William Howson, Derek J. Sloan, Aaron Mdolo, Catharina Boehme, Geoffrey A. Chipungu, Theresa J. Allain, Robert S. Heyderman, Elizabeth L. Corbett, David Alland

**Affiliations:** Malawi-Liverpool Wellcome Trust Clinical Research Program, University of Malawi College of Medicine, Blantyre, Malawia; Institute of Translational Medicine, University of Liverpool, Liverpool, United Kingdomb; Division of Infectious Disease, Department of Medicine, New Jersey Medical School, University of Medicine and Dentistry of New Jersey, Newark, New Jerseyc; Liverpool School of Tropical Medicine, Liverpool, United Kingdomd; Department of Medicine, University of Malawi College of Medicine, Blantyre, Malawie; Institute of Global Health, University of Liverpool, Liverpool, United Kingdomf; Foundation for Innovative New Diagnostics, Geneva, Switzerlandg; Department of Pathology and Medical Laboratory Sciences, University of Malawi College of Medicine, Blantyre, Malawih; London School of Hygiene and Tropical Medicine, London, United Kingdomi

## Abstract

Tuberculosis (TB) remains a leading cause of death among HIV-infected adults, in part because of delayed diagnosis and therefore delayed initiation of treatment. Recently, the Gene-Xpert platform, a rapid, PCR-based diagnostic platform, has been validated for the diagnosis of TB with sputum. We have evaluated the Xpert MTB/RIF assay for the diagnosis of Mycobacterium tuberculosis bacteremia and investigated its impact on clinical outcomes. Consecutive HIV-infected adults with fever and cough presenting to Queen Elizabeth Central Hospital, Blantyre, Malawi, were recruited and followed up for 2 months. At presentation, three sputum samples were examined by smear, culture, and Xpert MTB/RIF assay for the presence of M. tuberculosis and blood was drawn for PCR with Xpert, for mycobacterial culture (Myco/F Lytic), and for aerobic culture. One hundred four patients were recruited, and 44 (43%) were sputum culture positive for M. tuberculosis. Ten were Xpert blood positive, for a sensitivity of 21% and a specificity of 100%. The 2-week mortality rate was significantly higher among patients who were Xpert blood positive than among those who were negative (40% versus 3%; multivariate odds ratio [OR] for death if positive, 44; 95% confidence interval [CI], 3 to 662). This effect persisted on assessment of the mortality rate at 2 months (40% versus 11%; OR, 5.6; 95% CI, 1.3 to 24.6). When screening uncomplicated patients presenting with a productive cough for pulmonary TB, Xpert blood offers no diagnostic advantage over sputum testing. Despite this, Xpert blood positivity is highly predictive of early death and this test rapidly identifies a group of patients in urgent need of initiation of treatment.

## INTRODUCTION

Tuberculosis (TB) remains a leading cause of morbidity and mortality in HIV-infected adults, yet it is frequently difficult to diagnose, especially when the focus of infection is extrapulmonary. Autopsy studies in sub-Saharan Africa have revealed a high frequency of undiagnosed disseminated Mycobacterium tuberculosis infection in HIV-infected patients (1–3). Several studies have also reported a high prevalence of M. tuberculosis bacteremia, a form of disseminated TB (4–9). The clinical presentation of M. tuberculosis bacteremia mimics other bloodstream infections (BSIs), making it particularly difficult to identify and treat. Therefore, diagnostic tests that allow the early identification of M. tuberculosis bacteremia are urgently needed.

In addition to the clinical importance of M. tuberculosis bacteremia, several studies have established the potential of blood for TB detection by both culture and nucleic acid amplification test (NAAT) (10–14). Blood is an attractive sample type for TB diagnosis, especially for patients who are unable to expectorate or have paucibacillary sputum loads. Blood could be especially useful for HIV-infected patients, given their higher likelihood of sputum culture-negative, disseminated, or extrapulmonary TB (10, 11). Blood can be more reliably collected than sputum and does not pose the same respiratory biohazard either at collection or on processing within the laboratory environment.

Previous NAAT-based M. tuberculosis bacteremia studies have had mixed results, with sensitivities of TB detection in peripheral blood of 2 to 55% (10–14). The Xpert MTB/RIF is a NAAT that has recently been evaluated for the diagnosis of TB with sputum. This platform provides highly sensitive and specific detection of M. tuberculosis directly in untreated sputum in less than 2 h (15), but the assay cartridges hold a volume of only 1 ml. To improve the sensitivity of detection in blood, a lysis and centrifugation protocol was developed to enable 20 ml of blood to be tested in a single Xpert MTB/RIF assay, as reported in the accompanying article (16). Proof-of-principle data obtained with blood spiked with BCG suggested that this protocol could detect M. tuberculosis in blood with limits of detection of ≥95% at 10 CFU/ml and 33% at 0.5 CFU/ml, which are in the range needed for detection of M. tuberculosis bacteremia in patients with HIV infection.

In this study, we prospectively evaluated the role of the Xpert MTB/RIF platform in the diagnosis of M. tuberculosis bacteremia in HIV-infected Malawian patients presenting to hospital with high clinical suspicion of TB. We investigated the diagnostic utility of blood, directly compared the NAAT with standard mycobacterial blood culture (Myco/F Lytic) for the diagnosis of M. tuberculosis bacteremia, and investigated M. tuberculosis bacteremia as a risk factor for early death.

## MATERIALS AND METHODS

### Study design and population.

An observational cohort study was designed to evaluate the Xpert MTB/RIF assay for the detection of M. tuberculosis in whole blood of HIV-positive, symptomatic adults, aiming to establish whether there is potential value in sampling blood to diagnose TB. We recruited patients with HIV infection, fever of greater than 38.5°C, and cough for at least 1 week in a setting with a high prevalence of TB who were therefore highly like to have TB.

### Setting.

Malawi is a sub-Saharan African country with a high prevalence of HIV (estimated at 17.4% in 2010) (17) and where the rate of death in the first 2 months of TB treatment remains high (18, 19). Queen Elizabeth Central Hospital (QECH) is a 1,000-bed teaching hospital in Blantyre, one of the two largest cities in Malawi, serving a population of approximately 1 million people. Currently, however, routine microbiological investigation of inpatients for TB is limited to microscopy of a set of two sputum smears for acid-fast bacilli.

### Study procedures.

Baseline demographic and clinical information was collected. All patients underwent a standard evaluation including HIV testing, CD4 cell counting, and blood culture with an aerobic bottle (BacT/Alert; bioMérieux). An additional 20 ml of blood was collected in acid-citrate-dextrose solution B (ACD-B) tubes for the Xpert MTB/RIF assay, and a further 10 ml was collected for mycobacterial blood culture in Myco/F Lytic bottles. Three sputum samples were obtained (spot, morning, spot), two for standard smear and mycobacterial culture and one for Xpert MTB/RIF testing. Third-generation Xpert MTB/RIF cartridges were used, which have European Union Conformité Européenne *in vitro* diagnostic approval but which are not yet approved in the United States. The treatment of patients diagnosed with TB was in accordance with Malawi national guidelines (20). All patients were followed up by the study team at 2, 4, and 8 weeks.

### Case definitions.

The following case definitions were used: first, bacteriologically confirmed TB indicated by (i) two or more positive sputum smears (from three specimens), (ii) positive growth of M. tuberculosis in one or more sputum (two specimens) or blood (one specimen) cultures, or (iii) a positive sputum Xpert MTB/RIF result (one specimen); second, treated unconfirmed TB, i.e., patients without bacteriologically confirmed TB treated on the basis of clinical or radiological suspicion; and third, not TB, i.e., patients who were culture and Xpert MTB/RIF negative and not treated for TB.

### Laboratory methods.

All aerobic blood culture isolates were identified by standard diagnostic techniques (21). Organisms routinely found as part of the normal skin or oral flora were considered to be contaminants, including coagulase-negative staphylococci, diphtheroids, Bacillus species, and alpha-hemolytic streptococci other than Streptococcus pneumoniae (after clinical consideration of endocarditis). Antibiotic susceptibility testing was performed by the disc diffusion method according to British Society of Antimicrobial Chemotherapy standards (22).

Smears made from both direct and concentrated sputum samples were examined by iLED fluorescence microscopy (auramine O), with any positive results confirmed by Ziehl-Neelsen (ZN) staining. Sputum samples used for mycobacterial culture were decontaminated with 3% NaOH for 15 min and concentrated by centrifugation before inoculation of the resuspended pellet into mycobacterial growth indicator tubes and Lowenstein-Jensen (LJ) medium for up to 6 and 8 weeks, respectively. Mycobacterial isolates were further verified as M. tuberculosis or nontuberculous mycobacteria by microscopic cording and MPT-64 lateral-flow assays (Capilia; TAUNS Laboratories, Inc., Numazu, Japan) or, if either test was negative, growth on *p*-nitrobenzoic acid at room temperature and 45°C (23). The third sputum specimen was processed for the Xpert MTB/RIF assay in accordance with the manufacturer's recommended protocol (Cepheid, Sunnyvale, CA).

For mycobacterial blood culture, venous blood (5 ml) was inoculated into 50 ml broth (Bactec Myco/F Lytic; BD Microbiology Systems, Sparks, MD) and incubated at 37°C. Bottles were inspected daily for the first 14 days and then once every 2 days with a handheld UV Woods lamp. Contents of bottles were concentrated by centrifugation (3,000 × *g* for 20 min) either within 48 h after the first detection of fluorescence or at the end of 6 weeks of incubation (whichever occurred first). The concentrate was examined by ZN and Gram staining to exclude bacterial contaminants and subcultured on LJ medium. ZN stain-positive subcultures were then verified by lateral-flow assay as described above.

### Xpert MTB/RIF blood assay procedure.

The protocol described in the accompanying report was followed (16). In brief, of the 20 ml of blood drawn into four ACD-B tubes and inverted in accordance with the manufacturer's instructions, 18 ml was pooled, added to a centrifuge tube with lysis buffer, inverted, and centrifuged at 4,000 rpm (3,000 × *g*) for 20 min. After the supernatant was discarded, the pellet was resuspended in 1 ml of phosphate-buffered saline (pH 7.2) to which 1 ml of Xpert MTB/RIF assay sample reagent (SR) was added, incubated at room temperature for 15 min, and loaded into the sample chambers of Xpert MTB/RIF assay cartridges. The remaining 2-ml aliquot of blood was stored. At the end of the study, positive blood samples and an equal number of negative samples were retested with the Xpert MTB/RIF assay by using 1 ml of blood and adding 1 ml of Xpert MTB/RIF SR to it in order to assess whether the machine was as sensitive with smaller amounts of blood. The laboratory technician was blinded to the samples to reduce bias.

### Data management and statistical analyses.

Data were collected onto standardized proformas and entered into a secure Microsoft Access database with an optical character recognition system (ORC; Cardiff Teleforms, Cardiff, United Kingdom). Comparisons between patient characteristics were done by analysis of variance (ANOVA) or the Kruskal-Wallis test for continuous parametric and nonparametric variables, respectively. Categorical variables were assessed by the chi-square (χ^2^) test. Analysis of risk factors for death was done by multivariate logistic regression, and results were expressed as odds ratios (OR) with 95% confidence intervals (CIs). Survival of different patient groups over 2 months of follow-up was depicted by a Kaplan-Meier survival plot. Multivariate analysis was performed on factors found to be significant by univariate analysis by logistic regression. Statistical analysis used STATA v12 (Stata Corporation, College Station, TX).

### Ethics.

Approval of this study was obtained from the University of Malawi College of Medicine Research Ethics Committee (COMREC number P.02/11/1030). Informed written consent was obtained prior to enrollment in the study.

## RESULTS

### Characteristics of cohort.

**Fig 1:**
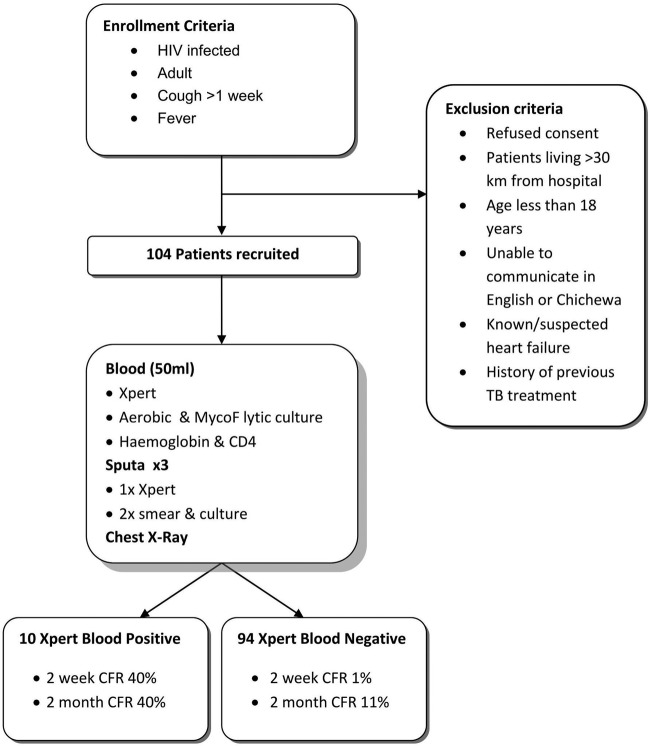
Flow chart of enrollment and exclusion criteria, study procedures, and survival analysis groups by Xpert MTB/RIF blood assay result. CFR, case fatality rate.

From August 2011 to November 2011, 104 HIV-infected adult patients meeting the inclusion criteria (fever and cough) were recruited (Fig. 1). Only 84 (81%) were able to produce a sputum sample before death or discharge, whereas all of the patients had blood drawn.

Fourteen (13%) participants had died by the end of 2 months of follow-up. Baseline characteristics are shown by vital status at 2 months in Table 1. The mean age was 37 years, and 68 (67%) participants were male. Patients reported a median duration of illness of 4 weeks. The mean hemoglobin level was 9.2 g/dl (*n* = 99), and the median CD4 cell count was 94/μl (*n* = 89, as there were 15 assay failures). Nineteen patients had a positive blood culture. M. tuberculosis was the most common isolate (*n* = 9), followed by S. pneumoniae (*n* = 6), nontyphoidal Salmonella (NTS; *n* = 3), and Haemophilus influenzae type b (*n* = 2). One patient was coinfected with both M. tuberculosis and Salmonella enterica serovar Typhimurium (Table 1). Univariate risk factors for death included male gender (*P* = 0.02) and M. tuberculosis bacteremia (*P* = 0.016).

**Table 1:** Baseline characteristics according to vital status at 2 months^*a*^

Baseline characteristic	Alive	Dead	*P* value
No. (%) of patients	90 (87)	14 (13)	
No. (%) of males	56 (62)	13 (93)	0.02
Age (yr)	37.1 (10.6)	35.7 (7.9)	0.63
Avg duration (weeks) of illness (quartiles 25–75)	4 (2–4)	4 (3–8)	0.73
No. (%) with hemoptysis	17 (19)	1 (7)	0.28
No. (%) with night sweats	59 (66)	7 (50)	0.79
No. (%) with wt loss	78 (87)	13 (93)	0.46
Avg systolic blood pressure (mmHg) (SD)	117 (57)	107 (17)	0.52
Avg respiratory rate (>28/min) (SD)	36 (42)	7 (50)	0.59
Avg Karnofsky score (quartiles 25–75)	70 (60–80)	70 (60–80)	0.79
No. (%) with hemoglobin level (g/dl) of:			
<7	13 (62)	7 (33)	0.03^*b*^
7–10	30 (80)	3 (8)	
10.1–13	29 (91)	2 (6)	
>13.0	8 (80)	0 (0)	
No. (%) with CD4 cell count/μl of:			
<50	18 (72)	4 (16)	0.26^*b*^
50–200	32 (84)	3 (8)	
>200	22 (81)	3 (11)	
Unknown	10 (71)	4 (29)	
No. (%) with BSI due to:			
Any pathogen	13 (72)	5 (28)	0.93
M. tuberculosis	5 (56)	4 (44)	0.016
Other	8 (89)	1 (11)	0.23
H. influenzae type b	1 (100)	0 (0)	
NTS	2 (67)	1 (33)	
S. pneumoniae	5 (100)	0 (0)	

a Two bacteremic patients were lost to follow-up by 2 months: 1 with H. influenzae type b, 1 with S. pneumoniae.

b *P* value for trend.

Eighty-two patients submitted three sputum samples, and a further two submitted two sputum samples. Forty-four patients (43%) met case definitions for bacteriologically confirmed TB, and all of them were sputum culture positive. There were no additional diagnoses of TB made by the Xpert MTB/RIF sputum assay or by mycobacterial blood culture in sputum culture-negative patients. No mycobacteria other than M. tuberculosis were isolated, and all samples positive by Xpert MTB/RIF assay (*n* = 33) were rifampin susceptible. Except for three invalid GeneXpert tests (4%) reported for sputum samples, no errors or sputum contamination events were observed.

All 104 patients were reviewed 2 weeks after discharge, and 95 were followed up until death or discharge from the study at 2 months. Follow-up visits ensured that patients had received the results of their investigations and had commenced antiretroviral therapy where indicated by Malawian national guidelines. In total, 7/104 (7%) had died after 2 weeks, 14/95 (15%) had died at 2 months, and 9/104 (9%) were lost to follow-up.

### Xpert-MTB/RIF assay for the diagnosis of TB from blood.

M. tuberculosis was detected by Xpert MTB/RIF assay in 10/104 samples (10%), with 66 (63%) negative and 28 (27%) invalid results or errors. Its sensitivity was 21% compared to that of the gold standard. Nine of these 10 patients were sputum culture positive; however, the 10th patient died before he could submit sputum; therefore, this patient was excluded from the sensitivity analysis. In contrast, only 4/9 (44%) Xpert blood-positive cases were sputum smear positive. The specificity of the Xpert blood assay was therefore 100% (95% CI, 94 to 100) (Table 2).

**Table 2:** Sensitivities and specificities of both the Xpert MTB/RIF platform with blood and conventional mycobacterial blood culture against the gold standard of sputum culture for TB diagnosis^*a*^

Test	No. positive/total, % sensitivity (95% CI)	No. positive/total, % specificity (95% CI)	No. positive/total, % PPV^*b*^ (95% CI)	No. positive/total, % NPV^*c*^ (95% CI)
Xpert MTB/RIF with blood	9/43, 21 (10–36)	61/61, 100 (94–100)	9/9, 100 (66–100)	60/94, 64 (53–74)
Blood culture	9/43, 21 (10–36)	61/61, 100 (94–100)	9/9, 100 (66–100)	60/94, 64 (54–74)

a The prevalence of TB in this study was 41%.

b PPV, positive predictive value.

c NPV, negative predictive value.

Although the sensitivity of 21% was identical to that of mycobacterial blood culture, there were some discrepancies between blood culture and the NAAT; 4/9 patients found to be Xpert blood positive were blood culture negative, while 4/9 patients who were blood culture positive were Xpert M. tuberculosis negative. Five patients were positive by both assays (56% concordance). The mean time to culture positivity was slightly less at 29 days for Xpert MTB/RIF-positive patients versus 34 days for Xpert MTB/RIF-negative patients.

On retesting of anticoagulated blood from participants who were Xpert blood assay positive, only 3/6 (50%) 1-ml aliquots were found to be positive, supporting the use of a lysis-centrifugation step to enable larger volumes of blood to be assayed by the Xpert platform (16).

### Characteristics of and clinical outcomes of M. tuberculosis bacteremia.

Of the 10 patients who were Xpert blood assay positive, 9 (90%) were male. Their mean hemoglobin level was significantly lower than that of the rest of the cohort at 6.5 g/dl (*P* = 0.0012), while their median CD4 cell count of 54/μl showed a trend toward being lower (*P* = 0.09). The 10 patients did not show a significant difference from the rest of the cohort at presentation in terms of age or duration of illness (Table 3).

**Table 3:** Comparison of clinical characteristics of patients in different diagnostic categories^*a*^

Diagnostic category or parameter	No. (%) of males	Mean age, yr (SD)	Mean illness duration, wk (SD)	Mean hemoglobin level, g/dl (SD)	Median CD4 cell count/μl (IQR)^*b*^
Xpert blood positive	9 (90)	38.1 (1.5)	3 (1.0–4.0)	6.8 (0.7)	54 (48–60)
PTB and Xpert blood negative	22 (63)	35.3 (2)	4 (2.0–8.0)	9 (0.4)	89 (29–373)
Treated, unconfirmed TB	21 (78)	37.1 (1.6)	4 (2.0–8.0)	9.2 (0.5)	108 (36–351)
Not TB	17 (53)	38.2 (2)	2.5 (1.0–12.0)	10.4 (0.5)	152 (23–490)
*P* value	0.11	0.71	0.82	<0.001	0.09

a Continuous parametric data (age, hemoglobin level, and duration of illness) were analyzed by ANOVA, continuous nonparametric data (CD4 cell count) were analyzed by Kruskal-Wallis test, and categorical data (gender) were analyzed by chi^2^ test.

b IQR, interquartile range.

**Fig 2:**
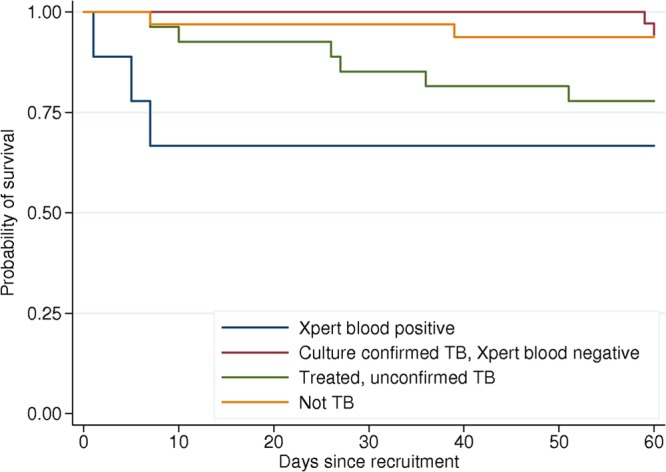
Kaplan-Meier survival plots by TB diagnostic group (Xpert MTB/RIF blood assay positive, culture-confirmed TB/Xpert blood assay negative, treated unconfirmed TB, and not TB).

The most striking association between Xpert blood results and clinical data related to patient outcome; 4/10 (40%) Xpert blood-positive participants died in the first 2 weeks (Fig. 2), with an adjusted OR of death of 44 (95% CI, 3 to 662) (Table 4). This effect persisted at 2 months, although the size of the effect was diminished (OR, 5.6; 85% CI, 1.3 to 24.6). Conversely, Xpert blood assay-negative participants with confirmed TB had a remarkably low mortality rate at 2 weeks (0/35 died) and 2 months (2/35 [6%] died). Of the four patients who were Xpert blood assay positive and died, three were positive by conventional mycobacterial blood culture; therefore, this effect was seen among TB blood culture-positive patients too; the OR of death with a positive blood culture was 11.4 (1.8 to 70.0) at 2 weeks and 6.8 (1.5 to 31.6) at 2 months.

**Table 4:** Univariate and multivariate analyses of risk factors for early death (2 weeks)^*a*^

Risk factor	OR (95 % CI)	
	Univariate analysis	Multivariate analysis
Xpert blood positivity	20.22 (3.66–111.78)	43.93 (2.92–661.55)
Low hemoglobin level	5.96 (1.34–26.58)	4.25 (0.51–35.68)
Low CD4 cell count	3.81 (0.83–17.51)	6.56 (0.36–120.20)

a Odds ratios per unit change in hemoglobin and CD4 count categories are shown.

## DISCUSSION

The main findings of this evaluation of the Xpert-MTB/RIF assay with blood samples from febrile HIV-infected patients suspected of having TB were that detection of M. tuberculosis bacteremia by NAAT was rapid and had high specificity (100%) but low sensitivity (21%) compared to sputum culture. Xpert-MTB/RIF blood results were highly predictive of early death, with 40% of the positive participants dying within the first 2 weeks of admission, compared to none of 35 participants with confirmed TB who were Xpert blood assay negative. The good prognosis of the Xpert blood assay-negative TB patients persisted to 2 months (2/35 [6%] died), despite a high prevalence of risk factors for death such as advanced immunosuppression, anemia, and WHO-defined clinical “danger signs.” Blood was tested with the Xpert-MTB/RIF assay only once, at presentation, because of the volume required; however, there was no evidence of occult M. tuberculosis bacteremia in the follow-up of this cohort and therefore no rationale for serial blood testing for M. tuberculosis.

In this study, the speed with which patients with M. tuberculosis bacteremia died was striking (Fig. 2). Unlike our study patients, people presenting to health care facilities in sub-Saharan Africa are often too sick or simply unable to produce sputum, making blood an attractive alternative sample for the diagnosis of TB. Furthermore, diagnostic facilities for febrile patients are frequently unavailable in sub-Saharan Africa; therefore, it is often normal to exclude or treat malaria first and then empirically treat bacterial infection and only after there is a failure to recover on antibiotics to empirically treat TB. Our data suggest that patients with M. tuberculosis bacteremia have a high risk of early death and that this incremental approach to empirical therapy introduces a potentially fatal delay in the initiation of anti-TB chemotherapy (ATC). Rapid testing of blood for TB with the Xpert platform may facilitate the rapid diagnosis of TB and consequently the early initiation of ATC.

The Xpert MTB/RIF blood test identified 100% of the patients who died of TB at 2 weeks and 67% (4/6) of those who died of TB at 2 months and so could potentially be used to distinguish patients requiring more intensive investigation and therapy from those who have a good prognosis under the current standard management approach. Of note, in the case of one of the two patients with pulmonary TB without M. tuberculosis bacteremia who died, the Xpert assay was not negative but failed. Also notable is that M. tuberculosis bacteremia accounted for a significant proportion of the deaths of febrile HIV-infected adults in this setting, irrespective of the final diagnosis (population-attributable fraction, 28% at 2 months postadmission).

Facilities were not available to induce sputum or perform bronchoalveolar lavage for patients who were unable to produce sputum or to perform postmortem examinations of all those who died before they could produce a sample. This was the first field test of the lysis-centrifugation protocol, which enables the concentration of large volumes of blood, and there were a number of “pressure-abort” failures caused by high pressures within the test cartridges, usually across the sample filter that is integrated into the cartridge. Visible clots were noted in many of the blood samples drawn. This was not noted in blood samples drawn from healthy volunteers in the U.S.-based study. Clots would logically be an obvious cause of high pressures. The clots in the blood suggest either an error in the technique used to draw blood or something more fundamentally different such as clotting factor activation associated with elevated cytokine levels because of systemic illness or dehydration. Further investigation is needed, but we suggest that blood with visible clots should not be tested with the Xpert platform.

Multiple studies from Africa with blood culture have demonstrated that TB is a common cause of BSI (24), and our data add to the existing evidence that it is an important cause of a high early death rate (25). Previous studies have not provided sufficiently timely data for clinical intervention, but the rapid diagnosis of M. tuberculosis bacteremia via the Xpert-MTB/RIF platform provides the opportunity to target patients at high risk of death. There is therefore an urgent need to investigate the conditions under which empirical or early treatment for M. tuberculosis sepsis should be commenced in order to prevent the high early death rate we have observed, starting with patients in regions with a high HIV prevalence who present with severe sepsis.
